# Natural magnetite as an effective and long-lasting catalyst for CWPO of azole pesticides in a continuous up-flow fixed-bed reactor

**DOI:** 10.1007/s11356-024-33065-8

**Published:** 2024-04-03

**Authors:** Neus Lopez-Arago, Macarena Munoz, Zahara M. de Pedro, Jose A. Casas

**Affiliations:** Chemical Engineering Department, Universidad Autónoma de Madrid, Ctra. Colmenar Km 15, 28049 Madrid, Spain

**Keywords:** Azole pesticides, Fixed-bed reactor, Magnetite, Decision 2022/1307, CWPO

## Abstract

The global occurrence of micropollutants in water bodies has raised concerns about potential negative effects on aquatic ecosystems and human health. EU regulations to mitigate such widespread pollution have already been implemented and are expected to become increasingly stringent in the next few years. Catalytic wet peroxide oxidation (CWPO) has proved to be a promising alternative for micropollutant removal from water, but most studies were performed in batch mode, often involving complex, expensive, and hardly recoverable catalysts, that are prone to deactivation. This work aims to demonstrate the feasibility of a fixed-bed reactor (FBR) packed with natural magnetite powder for the removal of a representative mixture of azole pesticides, recently listed in the EU Watch Lists. The performance of the system was evaluated by analyzing the impact of H_2_O_2_ dose (3.6–13.4 mg L^−1^), magnetite load (2–8 g), inlet flow rate (0.25–1 mL min^−1^), and initial micropollutant concentration (100–1000 µg L^−1^) over 300 h of continuous operation. Azole pesticide conversion values above 80% were achieved under selected operating conditions (*W*_Fe3O4_ = 8 g, [H_2_O_2_]_0_ = 6.7 mg L^−1^, flow rate = 0.5 mL min^−1^, pH_0_ = 5, *T* = 25 °C). Notably, the catalytic system showed a high stability upon 500 h in operation, with limited iron leaching (< 0.1 mg L^−1^). As a proof of concept, the feasibility of the system was confirmed using a real wastewater treatment plant (WWTP) effluent spiked with the mixture of azole pesticides. These results represent a clear advance for the application of CWPO as a tertiary treatment in WWTPs and open the door for the scale-up of FBR packed with natural magnetite.

**Supplementary Information:**

The online version contains supplementary material available at 10.1007/s11356-024-33065-8.

## Introduction

The worldwide occurrence of micropollutants in water resources represents a potential threat for aquatic ecosystems and human health (Figuière et al. 2022; Miyawaki et al. 2021). This vast array of anthropogenic compounds including pesticides, hormones, pharmaceuticals, personal care products, and industrial chemicals (Ahmed et al. 2017) is mainly introduced into the aquatic environment through wastewater treatment plants (WWTPs), which cannot warrant their complete elimination (Ben et al. 2018; García et al. 2021). With the aim to protect nature and human health from the most relevant micropollutants based on up-to-date scientific insights, the European Union (EU) has launched their control in EU water basins, urging its Member States to analyze the occurrence of the micropollutants included in the EU Watch Lists since 2015 (Decisions 2015/495, 2018/840, 2020/1161, and 2022/1307). These Watch Lists will be the basis for the upcoming review of the Urban Wastewater Treatment Directive, which will certainly limit the discharge of the most harmful substances. A particular case of hazardous micropollutants is the family of azole pesticides, included in the most recent EU Watch Lists (Decisions 2020/1161 and 2022/1307). These substances are increasingly applied as fungicides to control plant diseases, with a value share of 20–25% of the global fungicide volume market (Jørgensen & Heick 2021). Once in the aquatic environment, azole pesticides cause toxic effects on different living organisms like algae and fish (Chen & Ying 2015; Pesce et al. 2016; Poulsen et al. 2015; Storck et al. 2018).

The development of innovative but also highly effective, sustainable, inexpensive, and technically feasible water treatment systems that allow to create an absolute barrier to micropollutant emission at WWTPs represents a task of high priority nowadays. Advanced oxidation processes (AOPs), based on the in situ generation of strong and non-selective oxidizing radicals, appear as promising alternatives for such goal (Saravanan et al. 2021). Heterogeneous Fenton oxidation, also known as catalytic wet peroxide oxidation (CWPO), is particularly attractive as it combines the advantages of conventional homogeneous Fenton, *i.e.*, inexpensive chemicals, simple implementation, and mild conditions, with those of heterogeneous catalysis, *i.e.*, catalyst reusability and limited formation of iron sludge. In our previous contributions, we found that natural magnetite (Fe_3_O_4_), an inexpensive, sustainable, and highly available mineral, is an outstanding CWPO catalyst for the removal of a wide range of micropollutants included in the EU Watch Lists like macrolide antibiotics, hormones, diclofenac, neonicotinoid pesticides, and, very recently, azole pesticides (Lopez-Arago et al. 2023; Serrano et al. 2019, 2020). The latter were the lowest reactive towards CWPO, and thus, they are good candidates to be used as general indicators of the overall efficiency of the catalytic system.

The effectiveness of CWPO for micropollutant removal has been extensively demonstrated in a discontinuous operation context (*i.e.*, slurry batch reactors) due to the easiness of operation and the fast operational parameters screening, but the laborious recovery and recirculation of the powdered catalyst particles clearly hinders its practical implementation considering the large volumes of wastewater treated at WWTPs. These requirements represent the most important challenge to the state-of-the-art on CWPO, where continuous operation has been remarkably less investigated, and pilot plant studies are practically inexistent. A comprehensive review devoted to the application of continuous reactors (fixed bed, fluidized bed, and continuous stirred-tank reactors) in CWPO was developed by Esteves et al. (2016), where fixed-bed reactors (FBR) were pointed as attractive configurations as they allow amplifying the solid/liquid ratio and thus, accelerating the oxidation rate while reducing the contact times. Furthermore, when compared to the other continuous systems, the residence time is well controlled with minimum back mixing, and loss, as well as mechanical crushing of the catalyst, are avoided to a high extent.

Table 1 collects the most recent works (last 5 years) focused on the application of CWPO in continuous FBRs. As can be seen, iron oxide catalysts are the most relevant solids applied in this process. In particular, those based on synthesized magnetite have received major attention, which can be attributed to the presence of both Fe(II) and Fe(III) species, which significantly fasten the oxidation rate (Munoz et al. 2015). In general, catalysts preparation involves the use of synthetic organic or inorganic supports and requires relatively complex multi-step procedures like incipient wetness impregnation followed by high-temperature calcination (di Luca et al. 2018; Ding et al. 2020), combination of thermal treatments and metal–organic chemical vapor deposition (Yang et al. 2018), clay intercalation by auto-hydrolysis followed by calcination (Pinchao et al. 2021) or chemical co-precipitation and hydrothermal treatment (Huaccallo-Aguilar et al. 2021a, b; Huaccallo-Aguilar et al. 2021a, b). There are also attempts using different kinds of wastes as carbon support precursors like PET bottles (Thirumoorthy et al. 2021) or olive stones (Esteves et al. 2022), but in both cases, complex multi-step procedures and carbonization at high temperatures were required. Catalyst preparation clearly increases the cost of CWPO application, but what is even more important, synthetic catalysts usually suffer from deactivation. In fact, catalyst deactivation is one of the main concerns dealing with the application of CWPO in continuous mode. Leaching of iron, fouling of the catalytic surface, poisoning, and pore blocking are the main reasons behind this undesirable phenomenon (di Luca et al. 2018). As can be appreciated in Table 1, most reported catalysts suffer deactivation to some extent, being iron leaching as the most important cause for the activity loss. A metal-free catalyst based on a monolayer graphene film showed a reasonably good stability, but it exhibited a relatively low activity as denoted by the high operating temperature applied (80 °C) (Liu et al. 2020). On the other hand, it must be noted that most CWPO applications were focused on the treatment of aromatic compounds as well as dyes and industrial wastewaters at relatively high pollutant concentrations (mg L^−1^–g L^−1^). Studies focused on the abatement of micropollutants are scarce and carried out at pollutant concentrations clearly higher than the representative concentration at WWTP effluents (Huaccallo-Aguilar et al. 2021a, b; Huaccallo-Aguilar et al. 2021a, b). Furthermore, most works were performed at relatively high operating temperature (50–90 °C), and long-term studies are limited, with most publications testing time on stream of 1–3 days. All in all, there exists a noticeable knowledge gap regarding the development and application of robust and stable catalysts in CWPO in long-term operation. These studies are urgently required to demonstrate the feasibility of this system for potential application in WWTPs as tertiary treatment for micropollutant removal.

**Table 1:** Recent studies (2018–2023) focused on the application of CWPO in continuous FBRs

Catalyst	Target pollutant	Water matrix	Operating conditions	Main results (steady state)	Time on stream	Stability issues	Reference
Fe(III)-Al_2_O_3_ (6 wt% Fe)	Phenol	Deionized water	*C*_cont-H2O2_ = 1–6.1 g L^−1^ *W*_cat_ = 20 g *T* = 90 °C Flow rate = 1.2 mL min^−1^ *P* = 1 atm pH_0_ = 3	*X*_cont_ = 100% *X*_TOC_ = 90%	70 h	Iron leaching as main reason for deactivation. Attributed to chelating acidic intermediates Dissolved iron < 3 mg L^−1^ Cumulative iron loss = 20% of the initial load	di Luca et al. (2018)
Iron-loaded microfibrous entrapped carbon-nanotube catalyst (Fe_2_O_3_-MF-CNT) (0.6 wt% Cu)	m-cresol	Deionized water	*C*_cont-H2O2_ = 1–6 g L^−1^ *T* = 60 °C Flow rate = 6 mL min^−1^ *P* = 1 atm pH_0_ = 5	*X*_cont_ > 95% *X*_COD_ = 30%	24 h	Slight deactivation Low-iron leaching (quantitative results are not provided)	Yang et al. (2018)
Pelletized Cu catalysts (Cu/γ-Al_2_O_3_) (1.5 wt% Cu)	Methyl orange	Deionized water	*C*_cont-H2O2_ = 9.81–986 mg L^−1^ *W*_cat_ = 9.9 g *T* = 50 °C Flow rate = 2 mL min^−1^ *P* = 1 atm pH_0_ = 8.2	*X*_cont_ > 95% *X*_COD_ > 95%	200 h	Relatively high stability Dissolved copper = 0.4 mg L^−1^ Cumulative copper loss = 2.7% of the initial load	Ding et al. (2020)
Monolayer graphene film	Phenol	Deionized water	*C*_cont-H2O2_ = 1 –5.1 g L^−1^ *W*_cat_ = 4.8 g *T* = 80 °C Flow rate = 2 mL min^−1^ *P* = 1 atm pH_0_ = 6	*X*_cont_ = 100% *X*_TOC_ = 80–90%	72 h	High stability with steady conversion along the long-term experiment	Liu et al. (2020)
Al/Fe-pillared clay (0.5 wt% Fe)	Natural organic matter	Deionized water	*C*_TOC-H2O2_ = 10–472 mg L^−1^ *W*_cat_ = 27 g *T* = 20 °C Flow rate = 8 mL min^−1^ *P* = 1 atm pH_0_ = 7.2	*X*_TOC_ = 24%	30 h (3 h each run)	Dissolved iron < 0.15 mg L^−1^ Estimated cumulative iron loss = 1.6% of the initial load	Pinchao et al. (2021)
Fe_3_O_4_/multi-walled carbon nanotubes (25.4 wt% Fe)	Diclofenac and ibuprofen	Deionized water Surface water WWTP effluent Hospital wastewater effluent	*C*_diclofenac-ibuprofen-H2O2_ = 11.9–10.2–104.5 mg L^−1^ *W*_cat_ = 0.4 g *T* = 60 °C Flow rate = 1 mL min^−1^ *P* = 1 atm pH_0_ = 5	*X*_Diclofenac_ = 95% *X*_Ibuprofen_ = 90% *X*_TOC_ = 71%	20 h	Slight deactivation along 3 consecutive uses of 3 h time on stream Information about iron leaching not provided	Huaccallo-Aguilar et al. (2021b)
Fe_3_O_4_/multi-walled carbon nanotubes (25.4 wt% Fe)	Naproxen	Deionized water Surface water WWTP effluent Hospital wastewater effluent	*C*_cont-H2O2_ = 10–95 mg L^−1^ *W*_cat_ = 0.4 g *T* = 50 °C Flow rate = 1.7 mL min^−1^ *P* = 1 atm pH_0_ = 6.3	*X*_cont_ = 78% *X*_TOC_ = 83%	24 h	Iron leaching as main reason for slight deactivation Dissolved iron = 0.28 mg L^−1^ Cumulative iron loss = 15% of the initial load	Huaccallo-Aguilar et al. (2021a)
Al-doped magnetite spinel nanoparticles encapsulated in mesoporous carbon (21%γ-Fe_2_O_3_/28%FeAl_2_O_4_@MC)	Phenol	Deionized water	*C*_cont-H2O2_ = 0.2–1.2 g L^−1^ *W*_cat_ = 0.1 g *T* = 40 °C Flow rate = 0.1 mL min^−1^ *P* = 1 atm pH_0_ = 3	*X*_TOC_ = 80%	500 h	Relatively high stability despite metal leaching Dissolved iron = 1 mg L^−1^ (up to 2 mg L^−1^ along the first 200 h on stream) Dissolved aluminum = 2 mg L^−1^	Thirumoorthy et al. (2021)
Iron oxide supported on activated carbon prepared from olive stones (5 wt% Fe)	Phenolic compounds Real olive mill wastewater	Deionized water Real olive mill wastewater	*C*_cont-H2O2_ = 0.34–1.2 g L^−1^ (phenolic compounds) *C*_cont-H2O2_ = 1.25–2.75 g L^−1^ (olive mill wastewater) *W*_cat_ = 1 g *T* = 60 °C Flow rate = 0.75 mL min^−1^ *P* = 1 atm pH_0_ = 3.5 (phenolic compounds) pH_0_ = 4.5 (olive mill wastewater)	Phenolic compounds: *X*_cont_ = 87% *X*_TOC_ = 28% Real olive mill wastewater: *X*_cont_ = 57–71% *X*_COD_ = 26–34%	48 h	Iron leaching as main reason for deactivation Cumulative iron loss = 9% of the initial loss Full catalytic activity for 36 h	Esteves et al. (2022)
Natural magnetite	Azole pesticides mixture (tebuconazole, tetraconazole and penconazole)	Deionized water WWTP effluent	*C*_cont (each)-H2O2_ = 0.5–6.7 mg L^−1^ *W*_cat_ = 8 g *T* = 25 °C Flow rate = 0.5 mL min^−1^ *P* = 1 atm pH_0_ = 5	*X*_tebuconazole_ = 93% *X*_tetraconazole_ = 78% *X*_penconazole_ = 85%	500 h	High stability Dissolved iron < 0.2 mg L^−1^ Cumulative iron loss < 0.05% of the initial load	This work

The application of pristine magnetite mineral as catalyst could overcome the main shortcomings of synthetic catalysts in a unique manner as iron is part of the robust mineral structure, and thus, it is highly stable, with limited iron leaching (Munoz et al. 2018). Furthermore, its low surface area and practically negligible adsorption capacity for organic compounds would minimize possible poisoning, fouling, and pore blocking, as demonstrated in previous batch operation studies (Lopez-Arago et al. 2023; Serrano et al. 2019, 2020). Despite these advantages, to the best of our knowledge, pristine magnetite mineral has not been tested as a catalyst in continuous FBR so far.

The aim of this work is to demonstrate the feasibility of a FBR packed with natural magnetite powder for the CWPO of a representative mixture of azole pesticides (tebuconazole (TEB), tetraconazole (TET), and penconazole (PEN)) listed in the most recent EU Watch Lists (Decision 2020/1161 and Decision 2022/1307). This work clearly represents a significant advancement in the context of the literature, as it proves the viability of CWPO in long-term operation (500 h). The catalytic performance of the system was evaluated analyzing the impact of the main operating parameters, i.e., catalyst load, feed flow rate, hydrogen peroxide dose, and initial micropollutants concentration along 300 h on stream. To warrant the practical applicability of the catalytic system, all experiments were performed under ambient conditions. In the same line, a slightly acidic pH value (pH_0_ = 5) was established to maximize the H_2_O_2_ consumption efficiency, as demonstrated in our previous work in batch reactor operation (Munoz et al. 2018). Under optimized conditions, an additional long-term experiment for 200 h was performed to further demonstrate the high stability of the FBR packed with natural magnetite powder. As a proof of concept, a real WWTP sample fortified with the micropollutants mixture was finally employed as the inlet stream. These results represent an important step forward in the field of CWPO and open the door for its scale-up.

## Materials and methods

### Materials and chemicals

Azole pesticides (analytical grade quality), hydrogen peroxide solution (33% wt.), nitric acid (65%), and hydroxylamine (≥ 99%) were obtained by Sigma-Aldrich. Acetonitrile (99.9%) and 1,10-phenantroline (≥ 99%) were provided from Scharlau and Fluka, respectively. All reagents were used without further purification. The magnetite mineral powder employed as catalyst (ref: 500121500) was provided by Marphil S.L. (Spain). Natural magnetite granules (ref: 0029882796573), used to pack the magnetite powder in the FBR, were supplied by Inoxia (UK). Unless otherwise indicated, all trials were conducted using deionized water. The main properties of the azole pesticides tested in this work are summarized in Table 2.

**Table 2:**
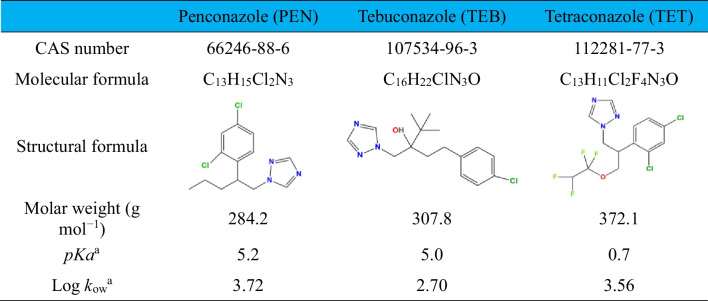
Main properties of the azole pesticides

### Catalyst characterization

The complete characterization of the powdered natural magnetite (Fe_3_O_4_) catalyst employed in this work can be found in our previous contribution (Munoz et al. 2018). Crystalline magnetite was the only phase present in the solid according to XRD analysis, being the content of iron similar to the theoretical one for pure magnetite (73 wt%). Consistent with these features, the solid showed strong magnetic properties (*M*_S_ = 77.9 emu g^−1^). The average size of the particles, which showed a rough spheric shape, was 0.2 µm, being the specific surface area value of 7 m^2^ g^−1^. The point of zero charge (pH_PZC_) was 7.8.

Furthermore, SEM images for both natural and used magnetite after 500 h of continuous operation were acquired using a JSM 6335F microscope (JEOL Ltd., Tokyo, Japan).

## Experimental procedure

**Fig. 1:**
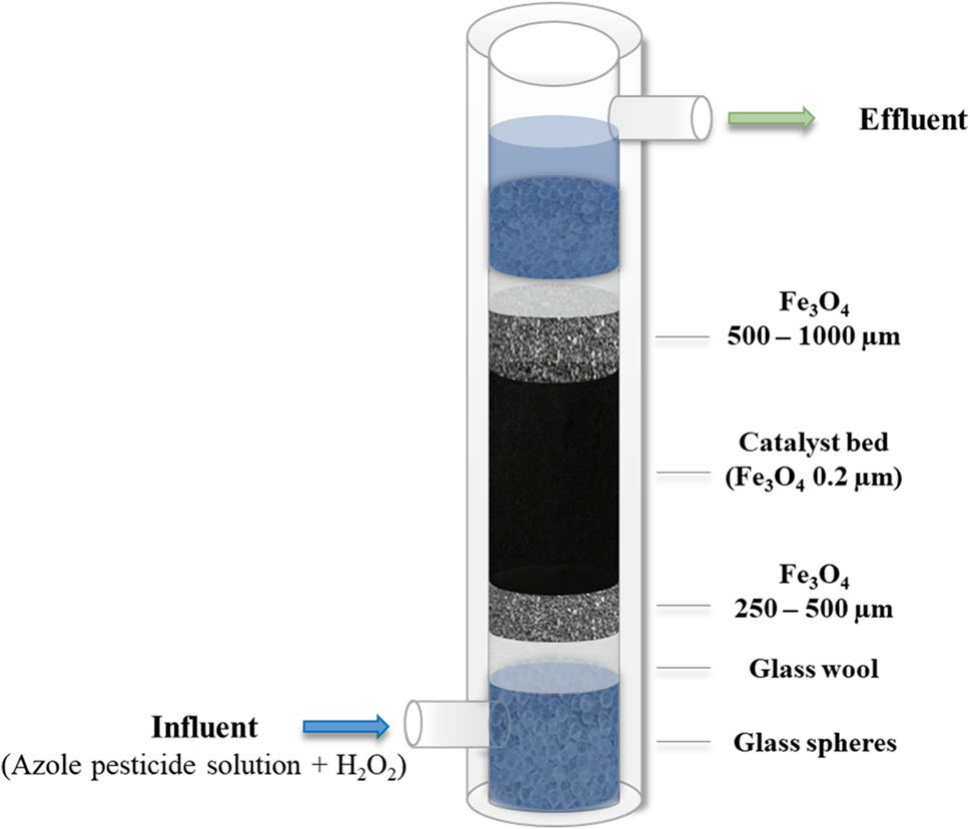
Scheme of the FBR packed with powdered magnetite catalyst used in the continuous CWPO experiments

The experimental setup used in this work is shown in Fig. 1. The FBR consists of a jacketed glass column (18 mm i.d., 115 mm length) where the catalyst bed (Fe_3_O_4_, 0.2 µm) was packed between two layers of magnetite granules. The bottom layer (Fe_3_O_4_, 1.5 g, 250–500 µm) was employed to improve aqueous solution dispersion in the catalytic bed. The top layer (Fe_3_O_4_, 6 g, 500–1000 µm) was added to prevent the possible loss of the fine catalyst powder taking advantage of the magnetic properties of both solids. These three layers were placed between two layers of glassy beads (2–3 mm) and a fine layer of glass wool. The reactor was continuously fed in up-flow mode using a peristaltic pump to prevent gas pocket formation and ensure that the catalyst powder is totally wetted.

CWPO trials were performed under ambient conditions (1 atm, 25 °C) at a slightly acidic pH value (pH_0_ = 5.0), which was adjusted with HNO_3_ (1 M). Operating temperature was kept constant by the upstream circulation of tempered water (25 °C) throughout the column jacket. The main parameters of the process were systematically investigated along 300 h on stream. The impact of each operating condition was assessed under steady-state conditions, and the experiments have an approximate duration of 24 h. Samples were taken from the reactor effluent every 1 h. Accordingly, the effect of catalyst weight in the bed (2–8 g), the reactor inlet flow rate (0.25–1 mL min^−1^), the hydrogen peroxide dose (3.4–13.4 mg L^−1^), and the initial pesticide concentration (100–1000 µg L^−1^) were studied. As depicted in Fig. 1, both the mixture of azole pesticides and H_2_O_2_ at the desired concentrations were transferred into the reactor in the up-flow inlet stream. A long-term continuous experiment (200 h) was accomplished after evaluating the operating conditions. Finally, the impact of the water matrix composition was also studied using a real WWTP effluent. All experiments were performed in triplicate, and the standard deviation was below 12%.

Preliminary experiments allowed to discard the possible role of azole pesticides adsorption on the catalytic bed, as the decrease on their concentration was negligible in the absence of H_2_O_2_. In the same line, it was also confirmed that H_2_O_2_ alone cannot efficiently oxidize the micropollutants under the operating conditions tested in this work (pesticide conversion < 5%). Finally, it was demonstrated that the magnetite granules used to pack the reactor were practically inactive for the CWPO reaction given their significantly lower exposed surface area compared to the fine catalyst powder.

### Analytical methods

Liquid samples were periodically taken from the reactor effluent along the continuous CWPO experiments. Azole pesticide concentration was determined by HPLC–UV (Shimadzu, Prominence-I model, LC-2030C LT) using an Agilent Eclipse Plus C18 column (15 cm length, 4.6 mm diameter) as stationary phase. A mixture of acetonitrile and ultrapure water at 0.8 mL min^−1^ was used as the mobile phase (60/40%, v/v). The detection wavelength was set at 222 nm for all the pesticides. Dissolved iron concentration was quantified by colorimetry with a UV 2100 Shimadzu UV–VIS spectrophotometer using the *o*-phenantroline method (Hoffman 1945). Total organic carbon (TOC) was determined with a TOC analyzer (Shimadzu TOC V_SCH_, Kioto, Japan).

## Results and discussion

### Kinetic study

**Fig. 2:**
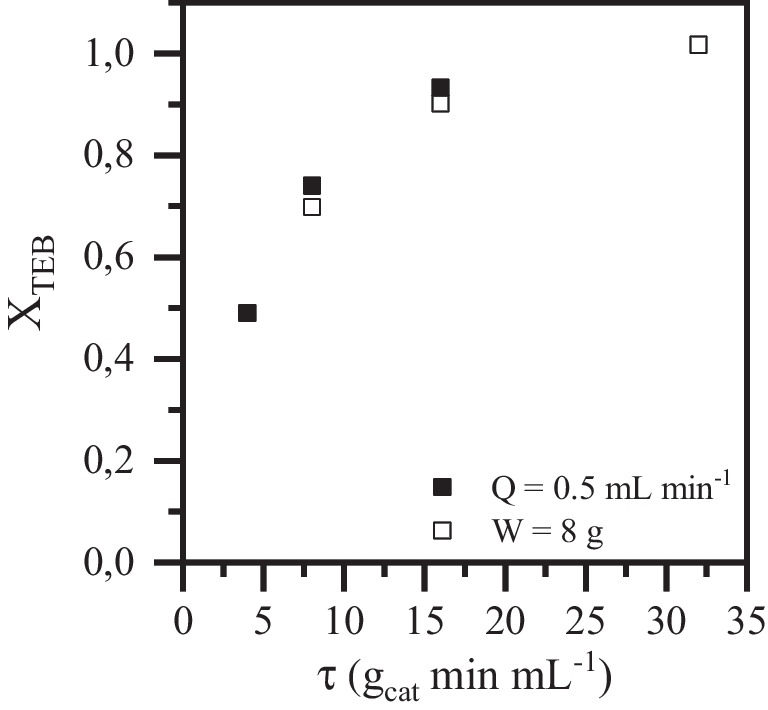
Effect of *τ* variations (4–32 *g*_cat_ min mL^−1^) on TEB oxidation ([TEB]_0_ = 500 µg L^**−**1^; *W* = 2–8 g; *Q* = 0.25–1 mL min^**−**1^; [H_2_O_2_]_0_ = 2.7 mg L^**−**^.^1^; pH_0_ = 5; *T* = 25 °C)

The possible existence of mass transfer limitations in the FBR packed with magnetite was evaluated prior to conducting the kinetic study. This preliminary analysis is crucial as mass transfer limitations lead to a performance decrease, increasing the operating costs (Alalm et al. 2021). For such study, the azole pesticide tebuconazole (TEB) was selected as a target pollutant since it is one of the most widely used triazole fungicides (Stamatis et al. 2015), being frequently detected in WWTP effluents in the order of ng L^−1^ (Stamatis et al. 2010). Moreover, as it will be shown in the following sections, TEB exhibited the highest reactivity among the three micropollutants tested in this work, although all of them showed removal yields above 80% in the optimum conditions. To check external mass transfer limitations in the reaction system, both the flow rate (Q) and catalyst load (W) were systematically varied, leading to different space–time values $\left(\uptau = \frac{{\text{W}}}{{\text{Q}}}\right)$ between 4 and 32 *g*_cat_ min mL^−1^. The results obtained are collected in Fig. 2. The modification of the flow rate, keeping constant the catalyst load, is denoted by the solid symbols, while the variation of the catalyst load, keeping constant the flow rate, is denoted by the square ones. As can be seen, external mass transfer limitations can be discarded under the operating conditions tested in this work as the conversion of the pollutant was maintained practically unchanged for the same space–time when the catalyst load or the flow rate was varied.

To further confirm the absence of external mass transfer limitations in the FBR system, the Carberry number (Ca) was calculated for TEB, the pesticide which showed the highest reactivity. For such goal, Sherwood, Reynolds, and Schmidt numbers were used for the estimation of the liquid–solid mass transfer coefficient (*k*_TEB,s_) of the system (see Supplementary Material for details). The Carberry number represents the relationship between the observed reaction rate and the maximum external mass transfer rate (Eq. 1).

1 $$Ca=\frac{{{({-r}_{{\text{TEB}}})}_{obs}}{\prime}}{{k}_{{\text{TEB}},{\text{s}}} {a}_{{\text{v}}} {C}_{{\text{TEB}},{\text{s}}}}$$

where${{ \left(-{r}_{{\text{TEB}}}\right)}_{{\text{obs}}}}{\prime}$ is the observed reaction rate for TEB ($mg {L}^{-1} {min}^{-1}$), *k*_TEB,s_ is the corresponding liquid–solid mass transfer coefficient (m s^−1^) for the micropollutant, *a*_v_ is the volumetric external surface area of the catalyst particles (m^2^ m^−3^), and *C*_TEB,s_ is the concentration (mg L^−1^) of the micropollutant in the liquid phase.${{\left(-{r}_{{\text{TEB}}}\right)}_{{\text{obs}}}}{\prime}$ was obtained experimentally, while *k*_TEB,s_ was estimated using the Sherwood number (see Table S1 of the Supplementary Material). The obtained values for the Carberry number were far below 0.05 under steady-state conditions which confirmed the absence of mass transfer limitations at the operating conditions testing in this work (Vega et al. 2022).

The determination of the reaction kinetics was experimentally accomplished by varying the concentration of the pesticides in the inlet stream. As a representative example, it must be noted that the conversion values obtained by the FBR for TEB (*X*_TEB_) at different starting micropollutant concentrations (from 100 to 1000 µg L^−1^) were in the range of 93–97% (see Fig. S1 of the Supplementary Material).

Therefore, varying the initial concentration of the pesticide in the inlet stream did not cause any significant change in the conversion of the pollutant under steady-state conditions. Accordingly, the process can be described by a pseudo-first-kinetic-order model. Considering this aspect, the mass balance for a continuous fixed-bed reactor was applied to determine the apparent kinetic constant of the reaction (*k*_app_) following Eq. 2.

2 $$\frac{W}{{F}_{i,o}}={\int }_{0}^{{X}_{i}}\frac{{dX}_{i}}{{(-{r}_{i})}_{obs}}$$

where *W* is the catalyst weight (g), *F*_i,0_ is the mass flow rate of the pesticide fed to the reactor (mg min^−1^), *X*_i_ is the pesticide conversion value, and ${\left(-{r}_{{\text{i}}}\right)}_{{\text{obs}}}$ is the reaction rate of each pesticide (mg *g*_cat_^−1^ min^−1^). Since a pseudo-first-kinetic-order model was proposed to describe the reaction, Eq. 2 can be rewritten as Eq. 3:

3 $$\frac{W}{Q {C}_{i,o}}={\int }_{0}^{Xi}\frac{dXi}{{k}_{app} {C}_{i,t}}={\int }_{0}^{Xi}\frac{dXi}{{k}_{app} {C}_{i,0} (1-{X}_{i})}$$

where *Q* is the flow rate (mL min^−1^), *C*_i,0_ and *C*_i,t_ are the pollutant concentration in the inlet and outlet streams, respectively (mg mL^−1^).

By the integration of Eq. 3, the value of the *k*_app_ (mL *g*_cat_^−1^ min^−1^) can be obtained by plotting the experimental data according to Eq. 4:

4 $${\text{In}}\left(\frac{1}{1-{X}_{i}}\right)={k}_{app}\frac{W}{Q}$$

The *k*_app_ calculated values for PEN, TET, and TEB removal were 0.12 ± 0.03, 0.09 ± 0.04, and 0.17 ± 0.03 mL *g*_cat_^−1^ min^−1^, respectively, along the different space-times tested in this work (4–32 *g*_cat_ min mL^−1^). The apparent pseudo-first-order rate constants for the three azole pesticides under all the operation conditions tested in this work are collected in Fig. S2, S3, and S4 of the Supplementary Material.

### Impact of operating conditions on FBR performance

**Fig. 3:**
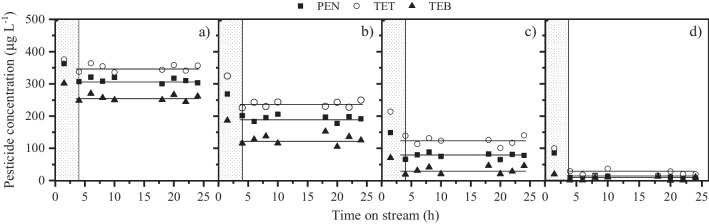
Impact of space–time (**a**: 4 *g*_cat_ min mL^**−**1^; **b**: 8 *g*_cat_ min mL^**−**1^; **c**: 16 *g*_cat_ min mL^**−**1^; **d**: 32 *g*_cat_ min mL^**−**1^) on the oxidation of the three azole pesticides ([TET]_0_ = [TEB]_0_ = [PEN]_0_ = 500 µg L^**−**1^; [H_2_O_2_]_0_ = 6.7 mg L^**−**^.^1^; pH_0_ = 5; *T* = 25 **°**C)

The impact of the space–time on the stability of the FBR system for pesticide removal was evaluated in the range of 4–32 *g*_cat_ min mL^−1^, varying both the flow rate and the catalyst load. The results obtained are depicted in Fig. 3. The shading area in the figure denotes the time required to achieve the steady state. In the first place, it must be noted that the azole pesticide reactivity decreased following the order: TEB > PEN > TET, which is consistent with our previous contribution where the removal of azole pesticides by CWPO in batch operation was investigated (Lopez-Arago et al. 2023). This phenomenon may be attributed to the competitive effects among different pesticides, which can be influenced by their chemical structure and properties (Table 2). TEB only contains a chlorine substituent at the *-para* position, while both TET and PEN contain two chlorine radicals attached to the aromatic ring in the *-orto* and *-para* position. The presence of halogen substituents stabilizes the delocalized electrons of the aromatic ring and thus, TET and PEN showed a lower reactivity than TEB. On the other hand, the main reason for the slower degradation rate of TET may be explained by the fact that it has less alkyl groups in its structure (Rokbani et al. 2019).

As observed in Fig. 3, the system showed a high stability since the micropollutant conversion remained practically unchanged along 25 h-experiments. Besides, iron leaching was below 0.1 mg L^−1^ in the FBR effluent in all cases. The removal of the target pollutants clearly increased with increasing the space–time. For instance, the conversion of TET, the least reactive pesticide towards CWPO, increased from ~ 30 to ~ 95% by varying the space–time from 4 to 32 *g*_cat_ min mL^−1^. Under these conditions, *i.e.*, a flow rate of 0.25 mL min^−1^ and a catalyst amount of 8 g, a residence time of around 30 min is assumed, considering that the reaction volume (useful volume) was ~ 8 mL. This residence time is quite attractive taking into account that 45 min is the average contact time in conventional adsorption tertiary treatment (Mailler et al. 2016).

**Fig. 4:**
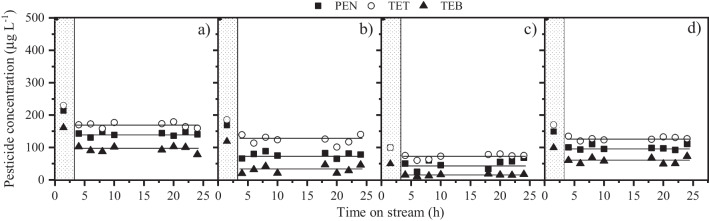
Impact of the H_2_O_2_ initial concentration (**a**: 3.6 mg L^−1^; **b**: 6.7 mg L^−1^; **c**: 10.0 mg L^−1^; **d**: 13.4 mg L^−1^) on the oxidation of the three azole pesticides ([TET]_0_ = [TEB]_0_ = [PEN]_0_ = 500 µg L^−1^; *τ* = 16 *g*_cat_ min mL.^−1^; pH_0_ = 5; *T* = 25 **°**C)

It is well-known that H_2_O_2_ represents the main operating cost in Fenton-based technologies and thus, the optimization of its consumption is a critical issue that must be considered (Berberidou et al. 2022; Cai et al. 2020). Although the impact of this operating cost is clearly more evident in the treatment of highly polluted industrial wastewaters (Munoz et al. 2014; Pliego et al. 2012) than in the degradation of micropollutants, any decrease in its intake clearly favors the economy of the process. Fig. 4 shows the results obtained in the continuous removal of azole pesticides by CWPO at different H_2_O_2_ doses. In particular, concentrations of 3.4, 6.7, 10.0, and 13.4 mg L^−1^ were tested, which approximately correspond to 50%, 100%, 150%, and 200% of the theoretical stoichiometric dose of H_2_O_2_ required to achieve the mineralization of the micropollutants. As observed, the conversion of the azole pesticides was slightly decreased when the stoichiometric dose of H_2_O_2_ was decreased by half. For instance, in the case of PEN, the conversion decreased from 85 to 71%. This result can be explained by the decrease in oxidizing species concentration under these conditions. Following the same trend, when the H_2_O_2_ dose was increased up to 150% of the theoretical stoichiometric amount, the conversion of the micropollutants was also increased. In this case, TEB conversion reached up to 97%. Nevertheless, a further increase of the H_2_O_2_ dose (up to 200% of the theoretical stoichiometric amount) led to a decrease of the azole pesticides conversion rate (e.g., TEB conversion was reduced to 88%). The concentration of H_2_O_2_ was then in clear excess so the oxidant itself could compete with the micropollutants for the available active sites at the catalytic surface, leading to an inefficient consumption of H_2_O_2_ and slower degradation of the pesticides (Farzaneh Kondori et al. 2018; Huaccallo-Aguilar et al. 2021a, b). Furthermore, H_2_O_2_ could also act as an autoscavenger for oxidizing radicals (mainly HO· and HOO·), leading to termination reactions, as the following ones (Eqs. 5–6) (Farzaneh Kondori et al. 2018; Pera-Titus et al. 2004):

5 $${{\text{H}}}_{2}{{\text{O}}}_{2}+{\text{HO}}\cdot\to {{\text{HOO}}}\cdot+{{\text{H}}}_{2}{\text{O}}$$

6 $${\text{HO}}\cdot+{\text{HOO}}\cdot\to {{\text{H}}}_{2}{\text{O}}+{{\text{O}}}_{2}$$

The apparent pseudo-first-order rate constant values also showed the same trend, obtaining the highest value at 150% of the theoretical stoichiometric dose of H_2_O_2_. For instance, the pseudo-first-order constant value obtained for the most reactive azole pesticide (TEB) was 0.22 mL *g*_cat_^−1^ min^−1^. Nevertheless, this value was quite similar to the one achieved with the theoretical stoichiometric dose of H_2_O_2_ (0.17 mL *g*_cat_^−1^ min^−1^) and thus, this dose was selected for further studies. Finally, it must be highlighted that the modification of the H_2_O_2_ dose did not lead to any significant change on the stability of the catalytic bed, being the dissolved iron concentration below 0.1 mg L^−1^ under the different conditions tested.

### FBR long-term stability

**Fig. 5:**
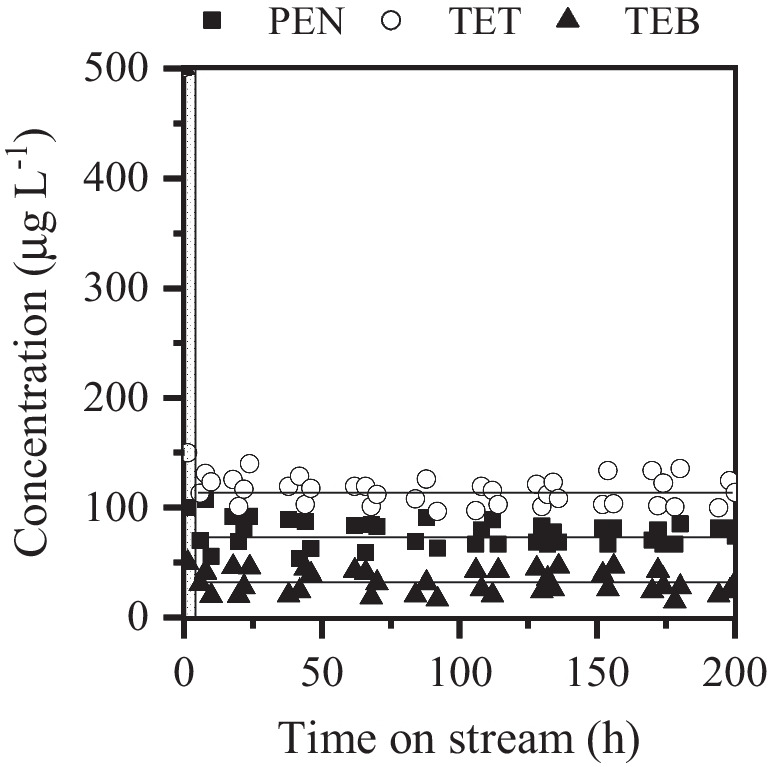
Long-term performance of the catalytic fixed-bed reactor upon CWPO of the three azole pesticides ([TET]_0_ = [TEB]_0_ = [PEN]_0_ = 500 µg L^−1^; *τ* = 16 *g*_cat_ min mL^−1^; [H_2_O_2_]_0_ = 6.7 mg L^−^.^1^; pH_0_ = 5; *T* = 25 **°**C)

**Fig. 6:**
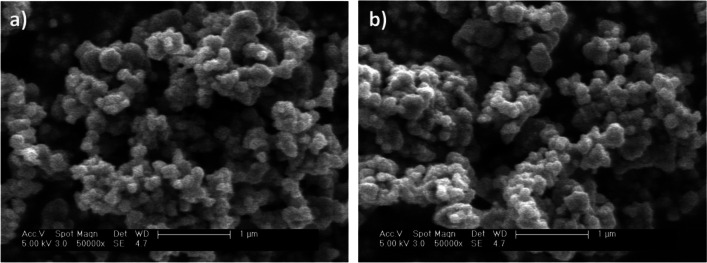
SEM images of fresh (**a**) and used (**b**) magnetite

To assess the stability of the system, a long-term continuous experiment was carried out for 200 h on stream under selected operating conditions (*τ* of 16 *g*_cat_ min mL^**−**1^, initial azole pesticides concentration of 500 µg L^**−**1^ and H_2_O_2_ dose of 6.7 mg L^**−**1^). As can be seen in Fig. 5, the FBR packed with magnetite showed an outstanding stability over 200 h on stream with no significant changes in the conversion values achieved for the azolic pesticides along the experiment. In fact, the main properties of the catalyst remained practically unchanged after being used. The absence of significant carbonaceous deposits on the surface of the used catalyst was confirmed, as the carbon content was determined to be less than 0.1 wt%. Additionally, it was observed that the specific surface area value and magnetic properties were practically the same as the measured for the fresh material. As a representative example, Fig. 6 shows a comparison between fresh (Fig. 6a) and used magnetite (Fig. 6b) SEM images after the long-term experiment. As can be seen, magnetite did not suffer any significant morphological change after 200 h of continuous treatment. Notably, the concentration of dissolved iron on the outlet stream was below 0.1 mg L^**−**1^. After the whole experiment, less than 0.05 wt% of the iron contained in the catalyst was leached.

### Proof of concept

The influence of the water matrix composition on the overall efficiency of the CWPO process is an important issue to be considered as the presence of co-existing substances could compete with the pollutants for the active sites of the catalyst, lead to the fouling of the catalyst surface, or act as oxidizing radical scavengers (Garcia-Costa et al. 2021; Miklos et al. 2018). To evaluate such impact, the treatment of a real WWTP effluent sample, spiked with the mixture of azole pesticides at 500 µg L^**−**1^, was evaluated. The main characteristics of the raw aqueous matrix are summarized in Table 3. The sample showed a relevant concentration of both organic and inorganic carbon sources. Furthermore, it also presented a non-negligible concentration of dissolved salts, being some of them common hydroxyl radical scavengers like chloride and sulfate (Lipczynska-Kochany et al. 1995; Lu et al. 2005; Siedlecka et al. 2007).

**Table 3:** Main characteristics of real water matrix tested

Parameter	Secondary WWTP effluent
pH	7.5
TOC (mg L^−1^)	6.3
IC (mg L^−1^)	12.1
Conductivity (µS cm^−1^)	1182
Cl^−^ (mg L^−1^)	21.4
NO_3_^−^ (mg L^−1^)	15.8
SO_4_^2−^ (mg L^−1^)	197
PO_4_^3−^ (mg L^−1^)	2.9

**Fig. 7:**
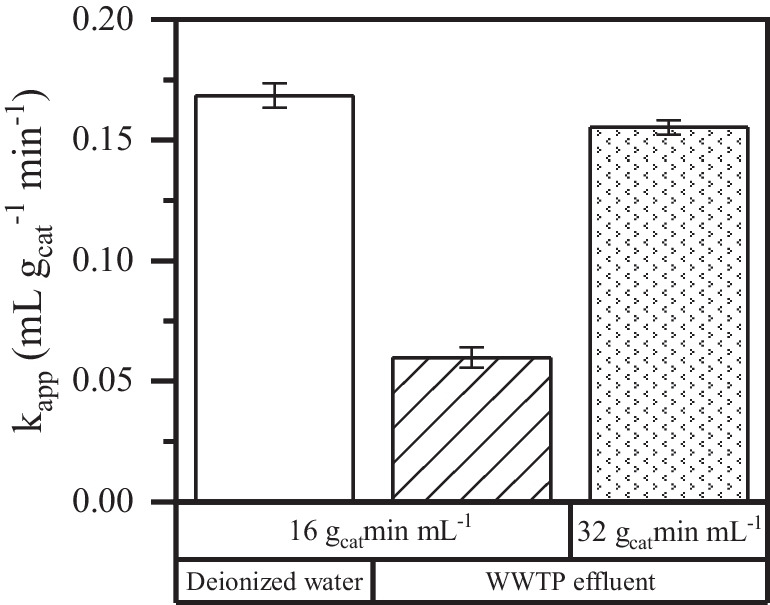
Apparent pseudo-first-order kinetic constant in the CWPO of TEB in different water matrix ([TEB]_0_ = 500 mg L^−1^; [H_2_O_2_]_0_ = 2.7 mg L.^−1^; pH_0_ = 5; *T* = 25 °C)

Given the relatively high organic matter load present in the real water matrix, the H_2_O_2_ concentration used in this experiment was 10 mg L^−1^, while the space–time was kept at 16 *g*_cat_ min mL^−1^. The results obtained are shown in Fig. 7 where the *k*_app_ of each experiment was obtained. For the sake of comparison, the results achieved using deionized water are provided as well. Clearly, the process was less efficient in the WWTP effluent, leading to a decrease of the pollutant conversion at the same space–time from 85%, 78%, and 93% to 62%, 48%, and 75% for PEN, TET, and TEB, respectively. The decrease in degradation efficiency of azole pesticides may be associated with the presence of organic and inorganic compounds in the treated water since they exhibit HO· scavenging properties (Bouanimba et al. 2015; Siedlecka et al. 2007). For example, the presence of salts such as bicarbonate or sulfate are well known to consume hydroxyl radicals, making them unavailable for pesticide oxidation. Even though sulfate and chloride radicals could be generated in these scavenging reactions, these radicals show a significantly lower oxidizing power than hydroxyl radicals (Dimitroula et al. 2012; Luo et al. 2014; Pignatello et al. 2006). On the other hand, the presence of organic species in the water matrix may cause competition with the pesticides for the active sites of the catalyst (Autin et al. 2013; Fan et al. 2013; Ye et al. 2019). To enhance the performance of the system and increase the pesticide conversion to at least 80% in the real WWTP effluent, the space–time was increased up to 32 *g*_cat_ min mL^−1^. Under these conditions, similar pseudo-first apparent kinetic constant values were obtained compared with the achieved with deionized water at a lower space–time (16 *g*_cat_ min mL^−1^). Consequently, by varying the space–time, comparable levels of conversion were obtained for the micropollutants when using deionized water. For instance, TEB exhibited a conversion of approximately 93% in deionized water, while a conversion of 91% was achieved using WWTP effluent. Furthermore, it must be noted that the system showed an outstanding stability, maintaining nearly consistent pesticide conversions throughout the 25-h experiment. In addition, the use of a real water matrix did not lead to carbonaceous deposits on the catalyst surface. Similarly, it was also verified that the use of a secondary effluent water matrix did not increase the concentration of dissolved iron compared to the use of ultrapure water (< 0.05 wt%).

## Conclusions

The FBR packed with natural magnetite has demonstrated to be highly effective and remarkably stable for the removal of a representative mixture of azole pesticides in continuous long-term operation. A comprehensive study was carried out to demonstrate the absence of mass transfer rate limitations and thus, to confirm that the system takes place under chemical kinetic control. The performance of the process was successfully described by a pseudo-first-order kinetic equation. The stability of the system was confirmed under long-term continuous operating, not appreciating any catalytic deactivation upon 200 h on stream. On the other hand, a complete operating condition study was carried out. It should be noted that variations in both space–time and H_2_O_2_ concentration led to changes in pesticide conversions, but in any case, the stability of the system was affected. For instance, the increase of *τ* led to a significant increase on the oxidation conversion of the pollutants up to > 95%, consistent with the higher residence time. Also, decreasing the stoichiometric H_2_O_2_ dose by half led to a decrease on the oxidation rate of the azole pesticides, while its increase by half led to a slight increase. On the opposite, a further increase of the H_2_O_2_ up to 200% of the stoichiometric amount caused a poorer performance, which can be explained by a competitive effect for the catalytic active centers and oxidizing radicals. All in all, azole pesticide conversion values above 80% were achieved under selected operating conditions (*W*_Fe3O4_ = 8 g, [H_2_O_2_]_0_ = 6.7 mg L^**−**1^, flow rate = 0.5 mL min^−1^, pH_0_ = 5, *T* = 25 °C). Finally, the versatility of the process was demonstrated using a real WWTP effluent spiked with the mixture of pesticides. The reaction matrix did not have any negative effect on the stability of the system although it led to a lower oxidation rate, which could be overcome by increasing the space–time. Considering the outstanding stability exhibited by the proposed catalytic system in this work, future research in this field should be focused on its scale-up to a pilot plant to yield valuable insights and substantiate the potential application of the catalytic system on a larger scale.

### Supplementary Information

Below is the link to the electronic supplementary material.

Supplementary file1 (DOCX 79 KB)

## Data Availability

Data will be made available on request.

## Abbreviations

CWPO: Catalytic wet peroxide oxidation
FBR: Fixed-bed reactor
WWTP: Wastewater treatment plant
EU: European Union
AOPs: Advanced oxidation processes
TEB: Tebuconazole
TET: Tetraconazole
PEN: Penconazole
XRD: X-ray diffraction
SEM: Scanning electron microscope
pH_PZC_: Point of zero charge
Log k_ow_: Octanol-water partition coefficient
TOC (mg L^−1^): Total organic carbon
IC (mg L^−1^): Inorganic carbon
Q (mL min^−1^): Flow rate
W (g_cat_): Catalyst load
τ (g_cat_ min mL^−1^): Space-time
Ca: Carberry number
(-r_i_)_obs’_ (mg g_cat_^−1^ min^−1^): Observed reaction rate
k_i,s_: Liquid-solid mass transfer coefficient
k_app_ (mL g_cat_^−1^ min^−1^): Apparent kinetic constant
a_v_ (m^2^ m^−3^): Volumetric external surface area
F_i,0_ (mg min^−1^): Mass flow rate
C_i,0_ (mg L^−1^): Pollutant concentration in the inlet stream
C_i_ (mg L^−1^): Pollutant concentration in the outlet stream

## Declarations

**Ethical approval.**

Not applicable.
