# Supplementary material for: Natural magnetite as an effective and long-lasting catalyst for CWPO of azole pesticides in a continuous up-flow fixed-bed reactor
Source: Environ Sci Pollut Res Int. 2024 Apr 3;31(20):29148–61. doi: 10.1007/s11356-024-33065-8 (PMC11058975; doi:10.1007/s11356-024-33065-8)
Supplement: Supplementary file 1 — Supplementary file1 (DOCX 79 KB) [file 11356_2024_33065_MOESM1_ESM.docx]

Natural magnetite as an inexpensive, effective, and long-lasting catalyst for CWPO of azole pesticides in a continuous up-flow fixed bed reactor

Neus Lopez-Arago*, Macarena Munoz*, Zahara M. de Pedro and Jose A. Casas

Departamento de Ingeniería Química, Universidad Autónoma de Madrid, Ctra. Colmenar km 15, 28049 Madrid, Spain

*Corresponding author phone: +34 91 497 4715 (N.L.), +34 91 497 3991 (M.M.); e-mail: [neus.lopez@uam.es](mailto:neus.lopez@uam.es); [macarena.munnoz@uam.es](mailto:macarena.munnoz@uam.es)

**Keywords:** azole pesticides; fixed-bed reactor; magnetite; Decision 2022/1307; CWPO.

1. **External mass transfer limitations study**

To ascertain the presence of external mass transfer limitations in the FBR system, the Carberry number (Eq. 1) was calculated for the reaction system when the most reactive compound, TEB, was oxidized:

$Ca=\frac{\left( {-r}_{TEB} \right)_{obs}´}{k_{TEB,s}a_{v} C_{TEB,s}}$ **(1)**

This number, when below 0.05, indicates the absence of external limitations impacting the system. To compute *Ca*, the $\left( {-r}_{TEB} \right)_{obs}$´ value was calculated considering a pseudo-first kinetic model, where *k_app_* was obtained for TEB in the kinetic study (0.17 mL g_cat_^-1^ min^-1^) and $C_{TEB,s}$was fixed at 0.5 mg L^-1^. To express the reaction rate in the proper units ($\left( {-r}_{TEB} \right)_{obs}´,mg L^{-1} \min^{-1}$), the density of the catalytic bed (831 g_cat_ L^-1^) was considered. Moreover, the volumetric external surface area of the catalyst particles (*d_p_* = 0.2·10^-6^ m) was calculated considering that they show spherical geometry ($a_{v}=30\cdot{10}^{6} m^{2}m^{-3}$).

The mass transfer coefficient (*k_TEB,s_*) was calculated from the Sherwood number (Eq. 2):

$Sh\boldsymbol{=} \frac{k_{TEB,s} d_{p}}{D_{m,TEB}}$ **(2)**

where$D_{m,TEB}$is the molecular diffusion coefficient of TEB in water at 25 ºC (m^2^ s^-1^) which value, according to Sarraute et al. (2019), is 0.36·10^-9^ m^2^ s^-1^.

To obtain the Sh number the following correlation, which is applicable across a broad spectrum of Reynolds number for a flow around spheres (Fogler, 1986) was used (Eq. 3):

$Sh=2+0.6{Re}_{p}^{1/2} {Sc}^{1/3}$ **(3)**

where Re_p_ and Sc are the Reynolds and Schmidt numbers, respectively.

**Table S1** shows the values obtained for the different calculated dimensionless numbers.

**Table S1**. Values of the different dimensionless numbers obtaining the Carberry number.

| $Reynold$  $number \left( {Re}_{p} \right)$ | ${Re}_{p}=\frac{v d_{p} \rho}{\mu}$ | ${Re}_{p}=7.3\cdot{10}^{-6}$ |
| --- | --- | --- |
| $Schmidt$  $number \left( Sc \right)$ | $Sc= \frac{\mu}{{\rho D}_{m,TEB}}$ | $Sc=2916.7$ |
| $Scherwood$  $number \left( Sh \right)$ | $Sh=2+0.6{Re}_{p}^{1/2} {Sc}^{1/3}$ | $Sh=2.0$ |
|  |  |  |
| $Carberry$  $number \left( Ca \right)$ | $Ca=\frac{\left( {-r}_{TEB} \right)_{obs}}{k_{TEB,s}a_{v} C_{TEB,s}}$ | $Ca=2.1\cdot{10}^{-8}$ |

1. **Kinetic study**

**Fig S1**. Impact of the initial pesticide concentration (a: 1000 µg L^-1^; b: 500 µg L^-1^; c: 100 µg L^-1^) on their oxidation (τ = 16 g_cat_ min mL^-1^; [H_2_O_2_]_0_ = 6.7 mg L^-1^; pH_0_ = 5; T = 25 ºC).

**Fig S2**. Apparent pseudo-first order kinetic constant obtained in the CWPO of PEN at different operating conditions. The azole pesticides were treated in a mixture (Standard conditions: τ = 16 g_cat_ min mL^-1^; [PEN]_0_ = 500 µg L^-1^; [H_2_O_2_]_0_ = 6.7 mg L^-1^; pH_0_ = 5; T = 25ºC).

**Fig S3.** Apparent pseudo-first order kinetic constant obtained in the CWPO of TEB at different operating conditions. The azole pesticides were treated in a mixture (Standard conditions: τ = 16 g_cat_ min mL^-1^; [TEB]_0_ = 500 µg L^-1^; [H_2_O_2_]_0_ = 6.7 mg L^-1^; pH_0_ = 5; T = 25ºC).

**Fig S4.** Apparent pseudo-first order kinetic constant obtained in the CWPO of TET at different operating conditions. The azole pesticides were treated in a mixture (Standard conditions: τ = 16 g_cat_ min mL^-1^; [TET]_0_ = 500 µg L^-1^; [H_2_O_2_]_0_ = 6.7 mg L^-1^; pH_0_ = 5; T = 25ºC).

**References**

Fogler, H. S., 1999. Elements of Chemical Reaction Engineering, third ed. Prentice-Hall International Edition, EE. UU.

Sarraute, S., Husson, P., Gomez, M.C., 2018. Effect of the diffusivity on the transport and fate of pesticides in water. Int. J. Environ. Sci. Technol. 16, 1857–1872. https://doi.org/10.1007/s13762-018-1815-7.
